# News and Miscellany

**Published:** 1904-01

**Authors:** 


### News and Miscellany.

DR. J. W. TALBOT, Texarkana, Texas, will receive for treatment and give personal attention to cases of morphine, cocaine and chloral addiction.

---

WILL REPAIR YOUR ELECTRICAL MACHINES.—Static and all electrical medical apparatus put in running order. I am also agent for electrical and X-ray apparatus. Oliver Brush, 710 Colorado Street, Austin, Texas.

---

NEW ORLEANS POLYCLINIC.—Seventeenth annual session opens November 2, 1903, and closes May 28, 1904. Physicians will find the Polyclinic an excellent means for posting themselves upon modern progress in all branches of medicine and surgery. The specialties are fully taught, including laboratory work. For further information, address New Orleans Polyclinic, postoffice box 797, New Orleans, La.

---

LUFKIN, TEXAS, December 9, 1903.

*Dr. F. E. Daniel, Austin, Texas.*

DEAR DOCTOR:—Enclosed find my check for $2, which is to settle up past dues, also to give me assurance for another year's subscription of the "Red Back" journal, which is, in my opinion, the cleanest and purest journal literature that has ever come to my notice.

Yours truly,

R. L. Denman.

---

Emergency Cases for Passenger Trains.—Mr. Ernest Hadra, of Dallas, son of the late eminent Texas surgeon, B. E. Hadra, has addressed a letter to the Texas railroad officials urging the carrying, on all trains, of a case supplied with such things as are needed by the surgeon and the wounded when a wreck occurs, (and wrecks have been epidemic this winter). The case should contain bandages, lint, sponges, antiseptics, anæsthetics, hemostatics, stimulants, adhesive plasters, liniments, forceps, needles, ligatures, tournequets, etc. Railroads have such things in their hospitals, of course, but it is sometimes hours before they can be gotten to the scene of the wreck, and many lives and much suffering could be saved if prompt assistance were rendered. The suggestion is eminently sensible; in fact, it should be required by law. It seems that some chief surgeon would have anticipated Mr. Hadra, and instituted the custom long ago.

---

For "looseness of bowels" I know nothing better than Listerine. It is "good for" many things,—sick stomach, vomiting, diarrhœa, cramps, and as an adjunct to the toilet, it is unsurpassed. Try some on your tooth brush. "No family should be without it."

---

As to Knopf.—Dr. Knopf, of New York, wrote a letter to the *Journal American Medical Association* (Dec. 12) decrying the American Congress on Tuberculosis, and also the imitation of same, the bolters congress (Lewis-Brown) and suggested that the real simon-pure article can only be organized by a convention of doctors which he proposes shall meet in Baltimore. The caller of the convention will doubtless organize it a-la-Knopf. Now, I am reliably informed that this Knopf wrote to the president of the American Congress of Tuberculosis, asking upon *what terms* he could get into the game. I will endeavor to get this letter or a copy of it for my February number. I have learned that Dr. Knopf has compiled some, but has never produced anv literature on consumption. Letter was not answered. Hence these tears.

---

An Ideal Tonic, Reconstructive in phthisis, bronchial affections and all nervous disorders is found in Phospho-Albumen (extract testes, spinal cord and brain). It is a rational remedy, and is based on sound therapeutic principles, and must appeal to the good sense and sound judgment of every conscientious physician. It is manufactured by the Phospho-Albumen Co., station M., Chicago, whose advertisement appears for the first time in the "Red Back." I advise my readers to thoroughly investigate this remedy.

---

Appreciation:—The Abbott Alkaloidal Co., Chicago., one of the livest and most successful manufacturing firms in America, and shrewd business men, generally, write me (December 30, '03): * * * "Continue our advertisement indefinitely. * * * We assure you that we are satisfied with the service, and it is a pleasure to instruct you to continue as above."

Many advertisers have been with me nineteen years.

Dr. W. S. Pickett, Karnes City, says: "Can't do without the "Red Back." All of which is most gratifying and shows that the "Red Back" is still booming and always keeps in the lead. Tra la!

---

My Dear Subscribers: I know that it is often very inconvenient to get a postal or express money order, and for so small an amount, many of your think, "it ain't worth while." Well, it *is* worth while to pay your subscription; so, just send me your personal check, and Jones—he'll pay the exchange.—Daniel.

---

It Gives me Pleasure to present to my readers, for the first time, the advertisement of the well-known and popular manufacturers of the staple remedy "Resinol," the Resinol Chemical Co., Baltimore, Md. Most of my readers have already made their acquaintance through their popular pharmaceuticals, and I know they will be glad to see them in such excellent company, in the "Red Back." I have none but the best and cleanest, all cash and prompt paying advertisers. I do not do a sample copy and chips and whetstone business. "Gentlemen, 'make you acquainted."

---

Victor Koechl & Co., the manufacturers of a special line of high class pharmaceuticals, and importers of others, and who are too well known to the medical profession to need any introduction at my hands, have an announcement in this issue of the "Red Back," and will have every month. I want all my readers to read it, and when ordering anesthesin, benzosol, orthoform, Knorr's antipyrin, or any of their specialties, tell them the "Red Back" said so.

---

Summer Course of Medical Lectures.—Boys, if you don't want to let up till next winter, go to Sewanee and finish this summer. Dr. Cain, the dean, is talking to you in this issue. See his announcement.

---

E. G. SWIFT, long connected with the house of Parke, Davis & Co., has been appointed general manager, vice, W. M. Warren, deceased.

---

I WANT to call attention to the advertisement of Dr. Leake's Infirmary, which appears in this issue of the "Red Back," and I want to say that if there is any doctor in Texas who is so unfortunate as to not know Leake, he should make a trip to Dallas especially to meet him. Most everybody knows him. His reputation as a gynecologist is extensive, and it has been sustained by uniform or nearly uniform, success in many of the gravest operations. In 1903 he lost not a case operated upon (and he had many of the capital gynecologic operations), and lost only one in 1902. Dr. Leake was a private pupil of Lawson Tait, and Tait's mantle rests worthily and gracefully on his shoulders.

---

DR. BACON SAUNDERS, of Fort Worth, was elected first vice president of the Southern Medical Association at the recent meeting at Atlanta, Ga.

---

DR. JNO. T. MOORE, of Galveston, who, during Dr. West's illness, has had charge of the office and papers of the secretary of the State Medical Association, and is familiar with the work, will fill out the unexpired year of Secretary West, ending with the April meeting, so I am advised by President Paschal. At that meeting a successor to the lamented West will have to be elected.

---

LOOK!—See the splendid showing of the "Red Back" advertising patrons. About ten new ones, and all the old and faithful ones have renewed for 1904, many for the twentieth round! If that is not a testimonial to the value of the "Red Back" as an advertising medium, what would be?

---

PERSONAL:—Mr. Tyree, the Chemist.—We are beginning with this issue an advertising contract with an old acquaintance of the doctors of Texas fifteen years ago, and I am sure many of them will remember him and his genial, gentlemanly nature and conduct. He was at that time representing a large manufacturing house in Philadelphia, and his annual visits were always a source of pleasure to Texas physicians who invariably profited by his intelligent conversations upon the subject of chemistry as applied to both pharmacy and medicine. The writer remembers him well, and the last conversation he had with him in which he stated that during all of his trips through Texas he had never found the latch string away from the door of a single doctor, and in severing his connection with detail work that there was no State in the Union he regretted so much to part with as Texas, for there was a plenty of good people, good eating, good climate and good liquor.

Since Tyree's retirement from detail work he has made a phenomenal success in commercializing in the United States and Europe what is known as Tyree's Antiseptic Powder, and he says that it is a source of great pride to him to know that he has never deviated from the strictest code of medical ethics in doing so. I hope that all of his friends in Texas will write to him and get a sample of his product, which I am sure would afford him much pleasure to send free of charge, for I have personally used it, and found it to be all he claims for it and more besides.

### Texas State Medical Association.

OFFICE OF THE PRESIDENT,

SAN ANTONIO, Texas, December 8, 1903.

*Dr. F. E. Daniel, Editor Texas Medical Journal, Austin, Texas.*

DEAR DOCTOR: I am anxious to get an expression of opinion from the medical profession of our State as to the measures that, in their opinion, should be considered by our association for its best interest, advancement and welfare.

I will esteem it a favor if you will kindly give space in your valued journal, and ask those who may feel inclined to do so, to write me fully, making such suggestions that they may think proper for the good of our association. I will study them, and in my annual message incorporate such as may be of practical value, and that may help in building up the profession and our association.

Thanking you in advance for bringing this matter to the attention of the members of our profession, I beg to remain,

Yours truly,

F. PASCHAL,

President State Medical Association.

